# Repetitive Immunosensor with a Fiber-Optic Device and Antibody-Coated Magnetic Beads for Semi-Continuous Monitoring of *Escherichia coli* O157:H7

**DOI:** 10.3390/s17092145

**Published:** 2017-09-19

**Authors:** Midori Taniguchi, Hirokazu Saito, Kohji Mitsubayashi

**Affiliations:** 1 Department of Biomedical Devices and Instrumentation, Institute of Biomaterials and Bioengineering, Tokyo Medical and Dental University, 2-3-10 Kanda-Surugadai, Chiyoda-ku, Tokyo 101-0062, Japan; QZG04715@nifty.ne.jp; 2 Department of Mechanical Engineering, Tokyo National College of Technology, 1220-1, Kunugida-machi, Hachioji-shi, Tokyo 193-0997, Japan; h.saito@tokyo-ct.ac.jp

**Keywords:** fiber-optics, fluorescence, evanescent wave, immunosensor, antigen-antibody complex, *E. coli*, magnetic beads

## Abstract

A rapid and reproducible fiber-optic immunosensor for *Escherichia coli* O157:H7 (*E. coli* O157:H7) was described. The biosensor consisted of a flow cell, an optical fiber with a thin Ni layer, and a PC linked fluorometer. First, the samples with *E. coli* O157:H7 were incubated with magnetic beads coated with anti-*E. coli* O157:H7 antibodies and anti-*E. coli* O157:H7 antibodies labeled cyanine 5 (Cy5) to make sandwich complexes. Then the Cy5-(*E. coli* O157:H7)-beads were injected into a flow cell and pulled to the magnetized Ni layer on the optical fiber set in the flow cell. An excitation light (λ = 635 nm) was used to illuminate the optical fiber, and the Cy5 florescent molecules facing the optical fiber were exposed to an evanescent wave from the optical fiber. The 670 nm fluorescent light was measured using a photodiode. Finally, the magnetic intensity of the Ni layer was removed and the Cy5-*E. coli* O157:H7-beads were washed out for the next immunoassay. *E. coli* O157:H7, diluted with phosphate buffer (PB), was measured from 1 × 10^5^ to 1 × 10^7^ cells/mL. The total time required for an assay was less than 15 min (except for the pretreatment process) and repeating immunoassay on one optical fiber was made possible.

## 1. Introduction

Bacteria detection is important in the food industry, clinical assay and environmental assessment. Conventional approaches for measuring bacteria cell counts employ a selective culture, serological and biochemical characterization. The achievement of sensitive and selective bacteria detection typically needs prolonged assay times (approx. 2 days) [1]. More sensitive and specific, and more simple and rapid approaches for bacteria detection are required for evaluating bacterial contamination.

In general, immunological assays are the methods that provide specific, reliable and reproducible detection of viruses and bacteria [2,3]. Conventional direct assays are RIA (radio-immunoassay) and ELISA (enzyme-linked immunosorbent assay). In both approaches (RIA and ELISA), an enzyme or radioisotope is covalently bound to the antibody or antigen. A sandwich immune-assay is a common method of ELISA or RIA. The unlabeled chemical is attached to a plastic well. The labeled antibody is permitted to bind to the unlabeled antigen. The antibody binding is measured as fluorescence by the product of an enzyme reaction in ELISA or as the amount of radioactivity by the coated well in RIA. Even though those approaches reduce the assay time compared with conventional culture methods, the RIA approach needs particular facilities, and ELISA needs cumbersome procedures. An alternative, microtiter plate methods, are used as plastic wells. However, the microtiter plate methods are complicated and time-consuming because of the wash processes and sample-preparations that are involved.

Fluorescent immunoassay (FLIA) with fluorescence dye for labeling antigens or antibodies has gained attention because there are no requirements such as particular facilities or enzyme reaction times [4]. Several types of biosensors have been developed by incorporating biological receptors and physical/chemical transducers. These biosensors represent a unique technology with potential for the rapid detection and continuous measurement of biomolecules [5,6]. An optical fiber immunosensor uses the FLIA principle. The sensor allows fluorescent measurement using an evanescent wave from a laser to detect an immune-complex on the surface of the optical fiber. This sensor can detect bacteria in foods by dipping the sensor fiber into the dominant area and allowing the real-time measurement of bacteria [7,8,9,10,11,12,13,14,15]. On the other hand, this kind of immnosensor needs a preparation process, involving immobilization to capture antibodies to the fiber-optic device surface (taking 14 to 22 h). Also, normal immunosensors are not adequate for repetitive immunoassay. If repetitive immunoassay was made possible, then serial measurement of bacteria, viruses or toxins in the blood, air or water could be achieved.

Alternatively, immune-magnetic separation techniques were successfully applied to isolate bacteria in recent research. The advantage of immune-magnetic separation is to separate directly targeted bacteria from samples by applying a magnetic field with no filtration and/or centrifugation [16,17,18,19,20,21]. In the research, we used the optical fiber immunosensor and immune-magnetic separation techniques to repeat measurement of the bacteria. We chose *Escherichia coli* O157:H7 (*E. coli* O157:H7) as the target bacteria. *E. coli* O157:H7 is strain of the bacterium *E. coli*. The strain has a powerful toxin and produces severe illness even with a low number of bacteria [22]. *E. coli* O157:H7 is needed not only for the rapid and easy appraoch, but also for the repetitive approach. The reproducible fiber-optic immunosensor for *E. coli* O157:H7 was developed by control of the captured antibodies’ attachment and release to the fiber optic surface by using antibody-coated magnetic beads and magnetic intensity.

## 2. Materials and Methods

### 2.1. Reagents and Bacteria

Serial dilution of a pure culture of *E. coli* O157:H7 (Positive Control, No. 50-95-90, Lot No. 030988, heat-treated, Kierkegaard and Perry Laboratories Inc., Gaithersburg, MD, USA) from 1.0 to 1.0 × 10^7^ cells/mL was prepared in a phosphate-buffer (pH 7.4, 10 mM) with 0.1% Tween20 (PBT) or commercially available milk. Non-pathogenic *E. coli* (formalin-treated, IAM12119), *Listeria monocytogenes* (formalin-treated, ATCC15313), *Vibrio* sp. (formalin-treated) (all provided by H. Endo, Tokyo University of Marine Science and Technology, Tokyo, Japan), was diluted to 1.0 × 10^5^ cells/mL to evaluate the sensor specificity.

A commercially available polyclonal antibody was used as a capture antibody (No. 01-95-90, Anti-*E. coli* O157:H7, Kierkegaard and Perry Laboratories Inc., Gaithersburg, MD, USA). An antibody label kit (PA35000, Cy5-Ab Labeling Kit, Amersham Biosciences, Buckinghamshire, UK) was prepared for labeling the antibody. The antibody was the same as the capture antibody (No. 01-95-90, Anti-*E. coli* O157:H7, Kierkegaard and Perry Laboratories Inc., Gaithersburg, MD, USA). The antibody was first added to a dye vial and incubated for 30 min at room temperature and mixed every 10 min. Next, free dye in the sample was removed using a gel filtration column from the labeling kit. The final concentration of the labeled antibody was measured by spectrophotometer to 0.64 mg/mL, and the final dye to antibody ratio was 7:1. The labeled antibodies were diluted with PB (1% BSA) and 0.1% Tween20 before use.

### 2.2. Assay Principle of Immunoassay with a Fluorometric Optical Fiber

**Figure 1:**
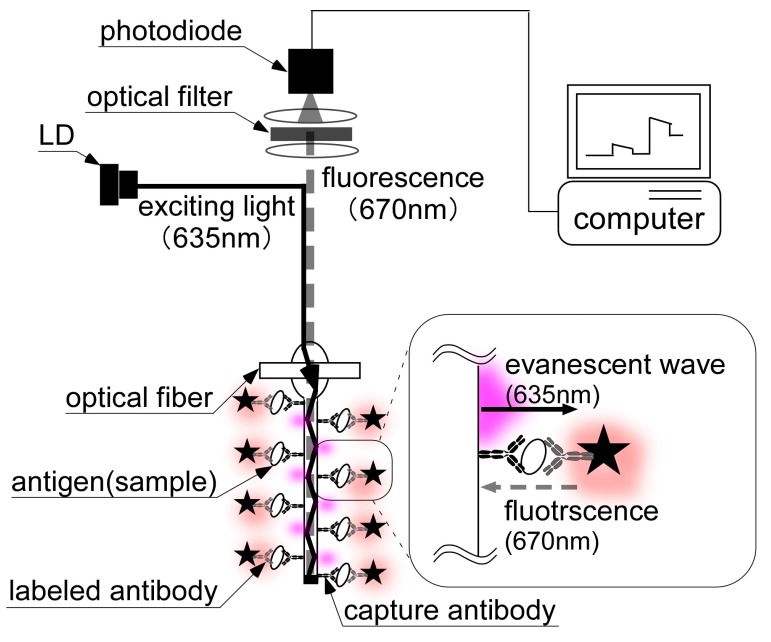
Assay principle of fluorescent immunoassay using optical fiber.

The assay principle of the immunoassay is shown in Figure 1. In order to detect *E. coli* O157:H7, a sandwich immunoassay was used with a capture antibody immobilized onto the fiber sensor and a Cy5-labeled antibody. An emitted light (λ: 635 nm) was illuminated to the proximal fiber end, and the Cy5 were excited with an evanescent wave. The molecule fluoresce (λ: 670 nm) was collected by the optical fiber, and measured using a photodiode.

### 2.3. Bacteria Measurement in Batch Phase

The optimum conditions for the optical fiber immune-assay for detection of *E. coli* O157:H7 in batch phase were investigated. First, the polystyrene fiber (4 cm and 0.78 mm) (Canon Inc., Tokyo, Japan) was incubated at 4 °C for 15 h with capture antibody (10 μg/mL, 100 μL). The optical fiber was rinsed with phosphate buffer (pH 7.4, 10 mM) and incubated with PB (100 μL, 1% of BSA) to minimize the nonspecific reaction.

Then, the optical fiber with capture-antibody was applied in a waveguide holder and washed with PBT. After that, PBT (350 μL) was injected into the holder. Then the proximal fiber end connected to a laser light source (635 nm) and the final reading was taken at the wavelength (670 nm) for 20 s (initial value: step 1). This value was recorded in picoamperes (pA). As the next step, the fiber sensor was dipped into Cy5-labeled antibody (100 μL, 1.25 μg/mL) for 5 min. The optical fiber was placed in a waveguide holder again, rinsed with PBT and signal readings were taken for 20 s (evaluate non-specific reaction: step 1’). The optical fiber was dipped into the sample (10 mL) for 5 min, placed in a waveguide holder and signal readings were taken (step 2). Finally, the fiber was dipped to Cy5-labeled antibody (100 μL) for 5 min again, placed in a waveguide holder and signal readings were taken (secondary antigen-antibody reaction: step 3). The total time required for an assay was less than 15 min (excluding the pretreatment process).

### 2.4. Scanning Electron Microscopy

Binding of *E. coli* O157:H7 to fiber sensors coated with capture-antibody was analyzed using a scanning electron microscope (SEM). The optical fibers were blocked as described above, and incubated in *E. coli* O157:H7 1.0 × 10^7^ cells/mL for 5 min. The control fiber sensors were devoid of capture antibody. The fiber sensors were washed in 2.5% gluteraldehyde for 15 h, rinsed with PB, immersed in buffered 1% osmium tetroxide for 90 min, rinsed with distilled water, freeze-dried and coated with a gold layer. Finally, the optical fibers were examined using the SEM (Hitachi High-Tech Science Systems: S-3400N, Tokyo, Japan) at 10 kV.

### 2.5. System Improvement for Flow Cell

A flow measurement system was applied for more prompt and convenient immunoassay. The flow measurement system was constructed from the optical fiber with a flow cell and an injector with a sample loop. The optical fiber was same as that of the batch phase immunoassay, and was fastened onto the flow cell.

### 2.6. Cy5-E. coli O157:H7-Beads

At first, 20 μL of Anti-*E. coli* O157:H7 antibody-coated magnetic microbeads (Prod. No. 710.03, Dynabeads anti-*E. coli* O157, Diameter: 2.8 μm) were added to a micro-centrifuge tube with *E. coli* O157:H7 samples (1.0 mL). The mixture was agitated for 15 min. The complex of the antibodies coated on the bead-*E. coli* O157:H7 were separated by a magnet and rinsed once with PBT. Then, 100 μL of Cy5-labeled antibodies (25 μg/mL) were added to the complex of the antibodies coated on the bead-*E. coli* O157:H7 and agitated for 15 min. The antibody-coated bead—*E. coli* O157—Cy5-labeled antibody complexes (Cy5-*E. coli* O157:H7-beads) were separated using the magnet and rinsed once with PBT.

### 2.7. A Thin Ni Layer Formed on the Optical Fiber

To eliminate preparation of optical fiber (capture antibodies immobilization) and to make repetitive immunoassay possible, control of the capture antibodies’ attachment and release to the fiber-optic surface by using the antibody-coated magnetic beads and magnetic intensity were devised.

A thin Pt layer was pre-coated to the aspect of the optical fiber by sputtering apparatus (CANON ANELVA: EVP-41398, Degree of vacuum: 1.0 Pa, T/S: 152 mm, Time: 15 min, Discharge power: 50 W, Ar: 10 sccm, Kanagawa, Japan). Then, Ni was plated (Nickel plating bath, pH 3.8, Power current: 0.04 A, Time: 45 min).

### 2.8. Magnetic Bead Attach/Release Immunoassay System

**Figure 2:**
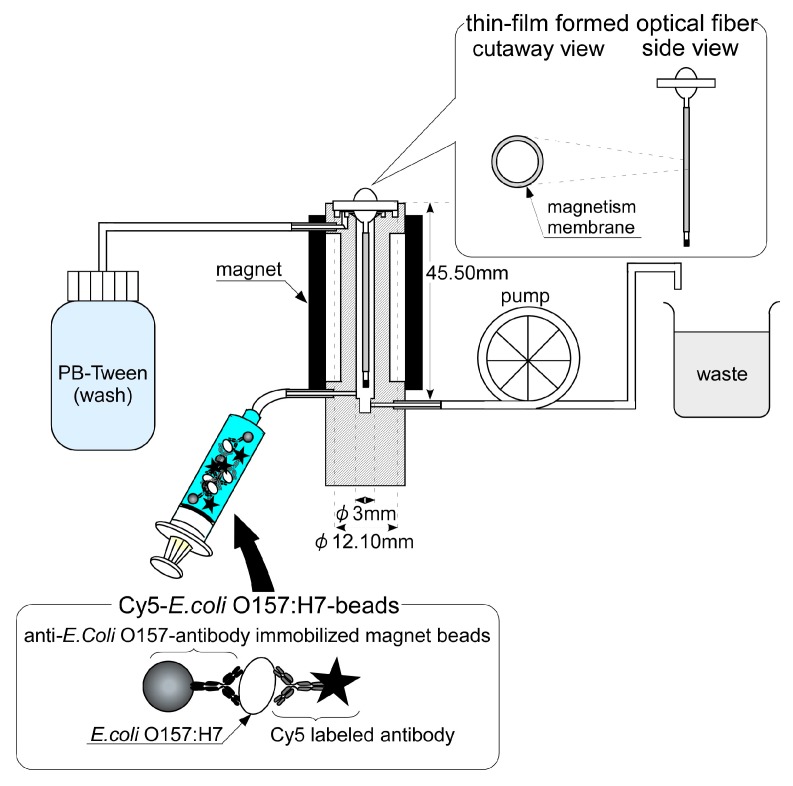
Pattern diagrams of the magnetic bead attach/release immunoassay system.

Using the flow cell, a magnetic beads attach/release immunoassay system was developed. The setup is shown in Figure 2. A thin Ni layer formed optical fiber was fastened onto the flow-cell and Cy5-*E. coli* O157:H7-beads were set in the injector.

First, the optical fiber with the thin Ni-film was rinsed with PBT. The proximal fiber end connected to laser light source and the final reading was taken at step A for 20 s. After that, magnets (neodymium magnet: 350 mT) were placed in the aspect of the flow cell and the Ni layer on the optical fiber was rendered magnetic. In this state, Cy5-*E. coli* O157:H7-beads were injected into the flow cell and collected by the magnetized Ni layer. The fluorometric intensity of this state was measured (step B). Then, PBT was injected into the flow cell and the optical fiber surface was rinsed. The fluorometric intensity of this state was also measured (step C). Finally, the magnets placed in the aspect of the flow cell were taken off, the magnetic intensity of Ni layer was removed, the Cy5-*E. coli* O157:H7-beads were washed out and the optical fiber was ready for the next immunoassay (step D (A’)).

## 3. Results and Discussions

The antibody-based fiber-optic immunosensor was a method developed to meet the need for sensitive and rapid microbial detection. Because of the promising applications of utilizing magnetic separation, magnetic-beads with bioactive molecules immobilized on the surface are attracting interest. We devised a reproducible fiber-optic immunosensor by controlling capture antibodies’ attachment to, and release from, the optical fiber surface. To control capture antibodies’ attachment and release from the optical fiber, we used antibody-coated magnetic-beads. First, we developed and evaluated an optical fiber immunoassay for *E. coli* O157:H7 in a batch phase.

### 3.1. Scanning Electron Microscopy Examination

**Figure 3:**
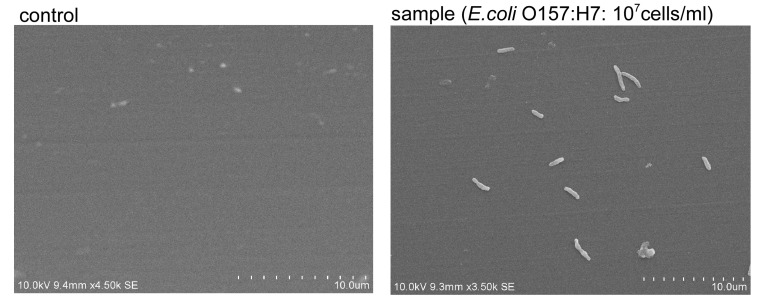
SEM images of optical fibers with captured *E. coli* O157:H7 cells. *E. coli* O157:H7 binding was examined on the aspect of the optical fiber. The left (control) is without capture antibody, and the right (sample) is with goat polyclonal capture antibody.

The optimum conditions for the optical fiber immunoassay of *E. coli* O157:H7 in a batch phase were considered. The binding of *E. coli* O157:H7 to fiber sensors coated using captured antibodies was assessed. SEM images are shown in Figure 3. As this figure indicates, the binding of *E. coli* O157:H7 to an optical sensor coated using captured antibodies was confirmed by the SEM analysis.

### 3.2. Batch Phase Immunoassay for E. coli O157:H7

**Figure 4:**
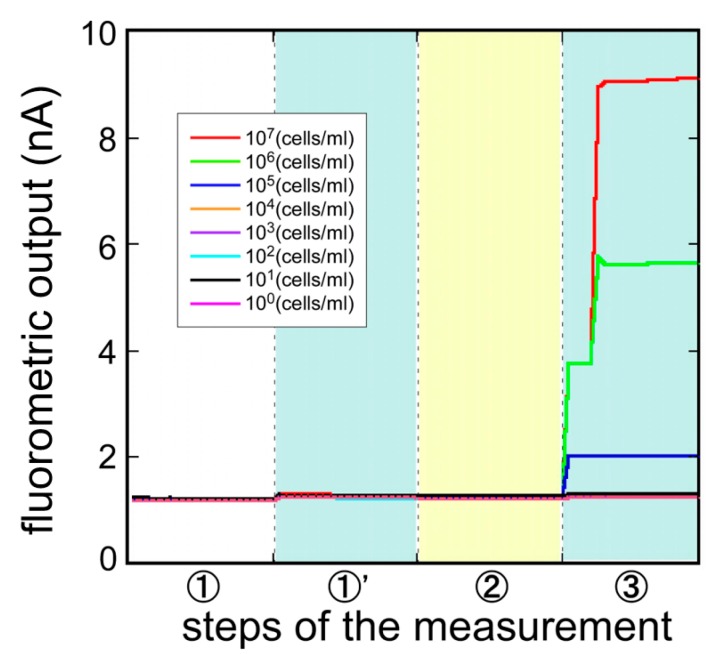
Typical responses of the immunosensor to varying concentrations of *E. coli* O157:H7.

The typical response of the batch phase immunoassay for *E. coli* O157:H7 in PB is shown in Figure 4. The signal difference between the primary (step 2) and secondary (step 3) antigen-antibody reactions was an output value. An *E. coli* O-157:H7 concentration of 1.0 × 10^7^ cells/mL gave the strongest signal enhancement (7838.3 pA) after secondary reaction. The signal strength decreased as the cell concentration decreased. The lowest concentration of cells that gave the signal (35 pA) compared to a control sample (no bacteria) was 1.0 × 10^3^ cells/mL and this was expected to be the detection limit for this immunosensor. There was almost no signal of non-specific reaction.

**Figure 5:**
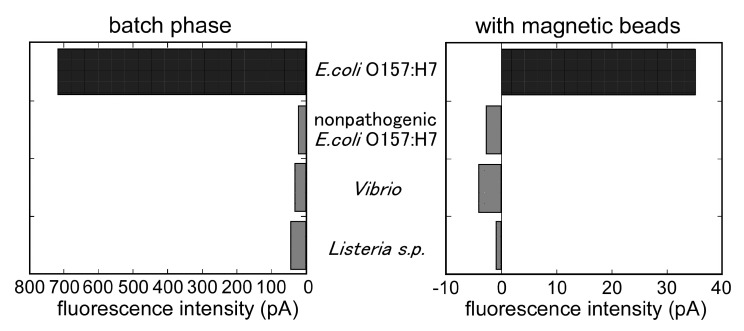
Signal comparison between *E. coli* O157:H7 and other bacteria. The left is the fluorometric output for each bacteria in the batch phase immunoassay. The concentrations of bacteria are 1.0 × 10^5^ cells/mL. The right is the fluorometric output for each bacterium in the magnetic bead attach/release immunoassay. (Concentrations of bacteria are 1.0 × 10^7^ cells/mL).

**Figure 6:**
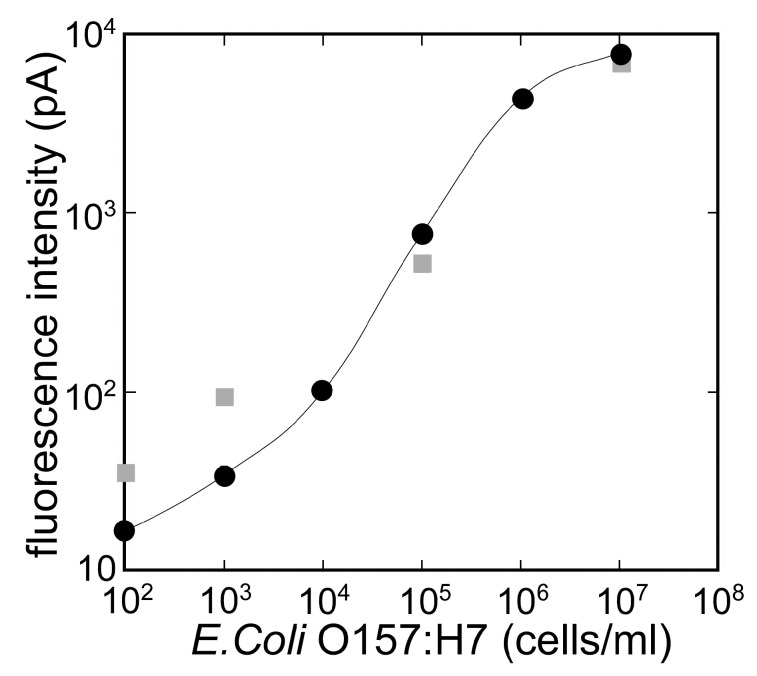
Calibration curve for *E. coli* O157:H7 in PBT (circle) and *E. coli* O157:H7 in milk (square).

Figure 5 shows the specificity of the immunosensor. The evaluation of the immuno-sensor with other bacteria indicated that *E. coli* O157:H7 (1.0 × 10^5^ cells/mL) generated an apparent signal of 783.1 pA, which was stronger than that of equivalent concentrations of *Listeria mono-cytogenes* (44.1 pA), *Vibrio* sp. (30.4 pA), and non-pathogenic *E. coli* (21.5 pA). The calibration curve for *E. coli* O157:H7 in PBT and *E. coli* O157:H7 in milk is shown in Figure 6. As the figure indicates, the output of the biosensor was related to the *E. coli* O157:H7 concentration. The immunosensor showed the reproducibility with a coefficient of variation: 11.8% at 1.0 × 10^5^ cell/mL of *E. coli* O157:H7 (*n* = 5). The lower detection limit of the immunosensor was 1.0 × 10^3^ cell/mL of *E. coli* O157:H7. Similarly, the output of the biosensor for *E. coli* O157:H7 in commercial milk was related to the *E. coli* O157:H7 concentration.

In the batch phase measurement, these results would be obtained in approximately 15 min of sampling. The detection limits for *E. coli* O157:H7 by ELISA could vary from 1.0 × 10^3^ to 1.0 × 10^5^ cells/mL [23,24] and the time for results could require from 1 to 2 h. The optical fiber immunosensor would be one of the best methods to allow for the sensitive, simple and rapid microbial detection. This approach could also detect *E. coli* O157:H7 in milk inoculated with 1.0 × 10^3^ to 1.0 × 10^7^ cells/mL. The calibration curve was similar to that of *E. coli* O157:H7 in a PBT solution. In general, fat globules and/or protein in milk caused some difficulties with filtering and the components are known to become in-activators for bacteria measurement. Being able to measure *E. coli* O157:H7 by dipping the optical sensor into milk means that this method could apply to the direct detection of bacteria in various kinds of food and drink samples.

On the other hand, this immunosensor needed to immobilize captured antibodies to the optical fiber surface before use, and the preparation of this took about 15 h. The batch phase immunoassay system needed reservoirs of the sample (*E. coli* O157:H7) and the Cy5-labeled antibody, and the immunoassay required migration of the optical fiber to the next reservoir after each reaction. Also, this kind of sensor is disposable but not adequate for repetitive or continuous immunoassay. And so the batch phase immunoassay system was improved to a flow cell immunoassay system. The flow measurement system was constructed using an optical fiber with the flow cell and the injector with the sample loop as shown in Figure 2. The optical fiber was fastened onto the flow cell. As a result, all the processes (rinse, reaction, measurement) were made possible in one flow cell. Therefore, extraneous magnetic intensity control became easy and a magnetic bead attach/release immunoassay system was devised.

### 3.3. Magnetic Bead Attach/Release Immunoassay

**Figure 7:**
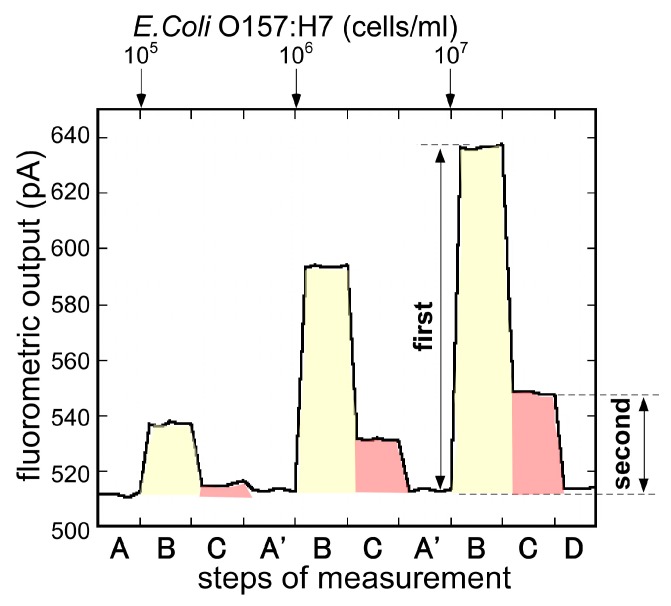
Typical response of magnetic bead attach/release measurement to the different concentrations of *E. coli* O157:H7 (1.0 × 10^5^, 1.0 × 10^6^, 1 × 10^7^ cells/mL). First output: signal differences between step A and step B (magnetic bead attach); Second output: signal differences between step B and step C (magnetic bead release).

Magnetic bead attach/release immunoassay was developed and evaluated. Figure 7 shows the typical responses of the biosensor measuring samples with 3 different concentrations of *E. coli* O157:H7 (1.0 × 10^5^, 1.0 × 10^6^, 1 × 10^7^ cells/mL) continuously. As the figure demonstrates, fluorometric output, dependent upon the concentration of *E. coli* O157:H7, was observed in step B and also in step C, when the surface of the Ni layer under the magnetization was rinsed. By removing the magnetization, the Cy5-*E. coli* O157:H7-beads were removed from the optical fiber surface and the fluorometric output recovered to the initial signal level, ready for the next immunoassay.

**Figure 8:**
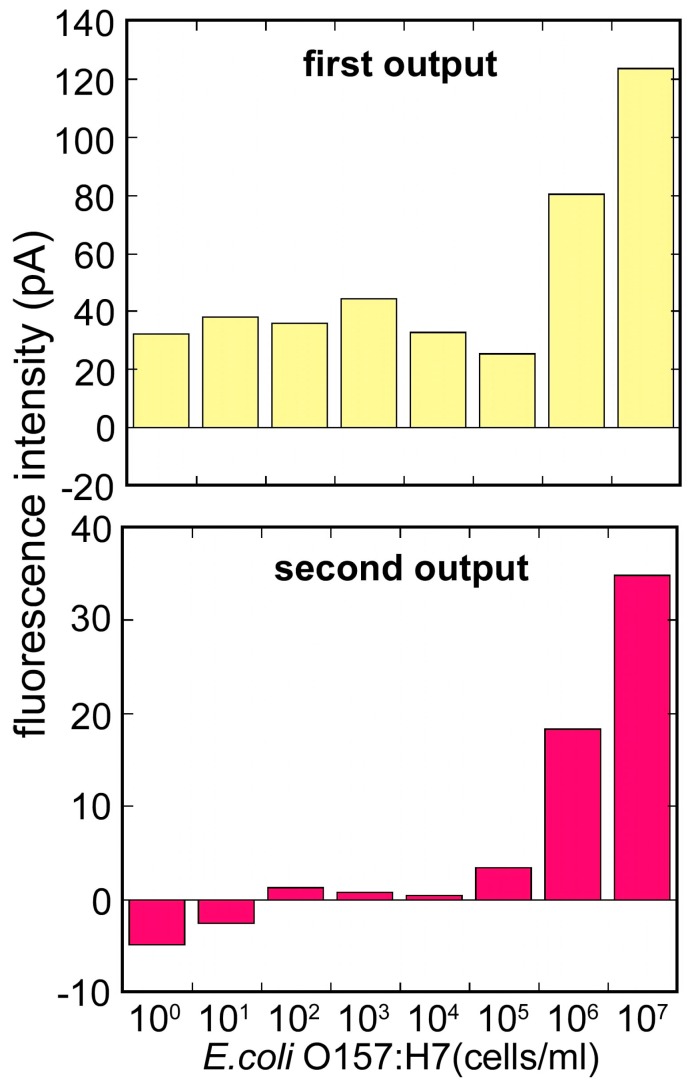
Comparison of the fluorescent intensity between [upper figure] the first (b/w Steps A and B: magnetic bead attach) and [lower figure] second outputs (b/w Steps B and C: magnetic bead release) to various concentrations of *E. coli* O157:H7 (1.0 × 10^0^–1 × 10^7^ cells/mL).

Signal differences between step A and step B were taken as the first output, and between step B and step C were taken as the second output, and the magnetic bead attach/release measurement was evaluated. Figure 8 compares the first output and the second output. In the first output, while the fluorometric intensity produced a high level of the noise, it seemed to be the effect of the free labeled antibodies. On the other hand, after the removal of the free labeled antibodies, the second output showed a low level of noise and an improvement of the signal to noise ratio. Using this measurement, it was possible to measure *E. coli* O157:H7 from 1.0 × 10^5^ to 1 × 10^7^ cells/mL. For the low concentration of *E. coli* O157:H7, the second output showed a plunge to sub-zero. It was maybe that the optical fiber surface condition changed, as the magnet beads attached to the optical fiber. To use the evanescent wave more effectively and achieve higher fluorescence intensity, further exploration of the Ni layer formation pattern and the layer thickness, and further exploration of the magnetic-beads size are need.

**Figure 9:**
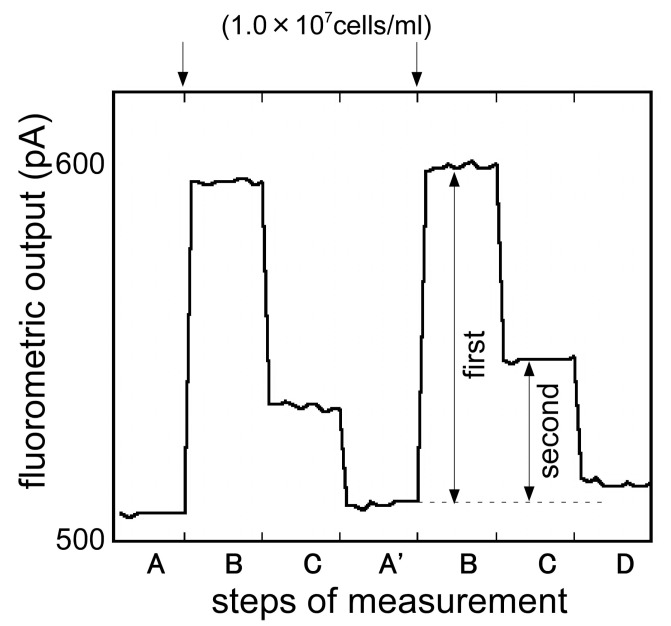
Typical responses of repetitive immunosensor to *E. coli* O157:H7 (1.0 × 10^7^ cells/mL).

**Figure 10:**
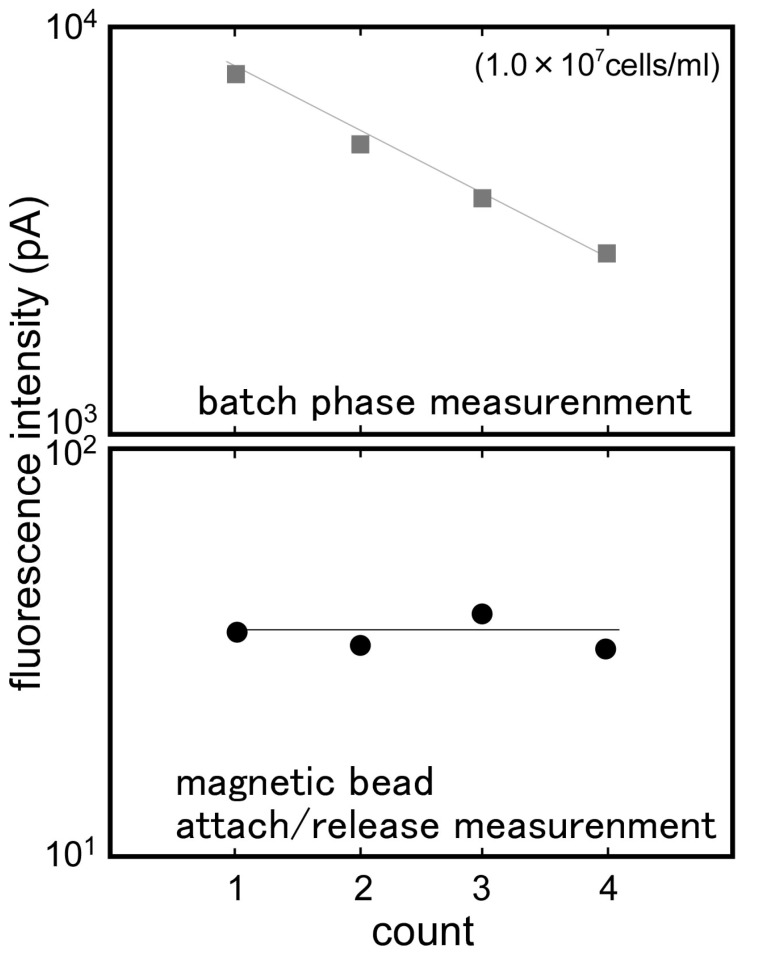
The parallel of the fluorometric output of the repetitive measurement using the same optical fiber between batch phase and magnetic bead attach/release measurements.

Figure 9 shows the typical response of repetitive measurement of *E. coli* O157:H7 (1.0 × 10^7^ cells/mL). Figure 10 shows the parallel of the fluorometric output of the repetitive measurement using the same optical fiber between the batch phase and magnetic bead attach/release measurements. In the batch phase measurement, the fluorometric output gradually decreased. It was probable that the free captured antibodies on the optical fiber surface decreased as the measurement time increased. On the other hand, the magnetic bead attach/release measurement indicated the possibility of the rapid recovery of the optical fiber surface, so repetitive measurement using the same optical fiber was possible. The detection time required for an assay was less than 20 s (expect for the sandwich complexes forming time) for one sample and it was available to measure a number of samples in a short timeframe.

The specificity of the sensor is shown in Figure 5. The evaluation of the optical fiber with other bacteria indicated that *E. coli* O157:H7 (1.0 × 10^7^ cells/mL) generated a signal (35 pA) that was stronger than that of an equivalent concentration of *Listeria mono-cytogenes* (−0.97 pA), *Vibrio* sp. (−2.72 pA), and non-pathogenic *E. coli* (−2.7 pA).

## 4. Conclusions

A fiber-optic immunosensor for semi-continuous immunoassay was constructed. It was based on a sandwich immunoassay, using captured antibody immobilized magnetic beads and a Cy5-labeled antibody for measurement. Prior to developing the semi-continuous immunoassay, the fiber-optic immunosensor in the batch phase was constructed and evaluated using an *E. coli* O157:H7 suspension. While the sensor needed to immobilize captured antibodies to the fiber-optic immunosensor surface before use, the calibration range for *E. coli* O157:H7 diluted in PBT was from 1.0 × 10^3^ to 1.0 × 10^7^ cells/mL. The optical immunosensor could also detect artificially inoculated *E. coli* O157:H7 in commercially available milk. The calibration curve was similar to that of *E. coli* O157:H7 in PBS. The results indicate that the immunosensor could directly detect *E. coli* O157:H7 in food/drink samples. In the batch phase, the optical sensor results were obtained in approximately 15 min of sampling.

To eliminate the preparation of the optical fiber and to make repetitive immunoassay possible, control of the captured antibodies’ attachment and release to the fiber-optic surface by using the antibody-coated magnetic beads and magnetic intensity were developed. The calibration range for *E. coli* O157:H7 diluted using PBT was from 1 × 10^5^ to 1 × 10^7^ cells/mL. The repetitive measurement using the same optical fiber was possible using the magnetic beads. The total time required for an assay was less than 15 min (excluding the time for the pretreatment process) for one sample and it was available to measure a number of samples in a short space of time. The semi-continuous fiber-optic immunosensor could contribute to many different areas such as the food industry, environmental monitoring, safety and health monitoring, and clinical testing.
